# TRAIP regulates replication fork recovery and progression via PCNA

**DOI:** 10.1038/celldisc.2016.16

**Published:** 2016-06-28

**Authors:** Wanjuan Feng, Yingying Guo, Jun Huang, Yiqun Deng, Jianye Zang, Michael Shing-Yan Huen

**Affiliations:** 1 School of Biomedical Sciences, LKS Faculty of Medicine, The University of Hong Kong, Pokfulam, Hong Kong S.A.R., China; 2 Centre for Cancer Research, LKS Faculty of Medicine, The University of Hong Kong, Pokfulam, Hong Kong S.A.R., China; 3 Life Sciences Institute, Zhejiang University, Zhejiang, China; 4 College of Life Sciences, South China Agricultural University, Guangzhou, China; 5 School of Life Sciences, University of Science of Technology of China, Hefei, China; 6 State Key Laboratory of Brain and Cognitive Sciences, The University of Hong Kong, Pokfulam, Hong Kong S.A.R., China

**Keywords:** RNF206, TRAIP, PCNA, replication, hydroxyurea, DNA damage

## Abstract

PCNA is a central scaffold that coordinately assembles replication and repair machineries at DNA replication forks for faithful genome duplication. Here, we describe TRAIP (RNF206) as a novel PCNA-interacting factor that has important roles during mammalian replicative stress responses. We show that TRAIP encodes a nucleolar protein that migrates to stalled replication forks, and that this is accomplished by its targeting of PCNA via an evolutionarily conserved PIP box on its C terminus. Accordingly, inactivation of TRAIP or its interaction with the PCNA clamp compromised replication fork recovery and progression, and leads to chromosome instability. Together, our findings establish TRAIP as a component of the mammalian replicative stress response network, and implicate the TRAIP-PCNA axis in recovery of stalled replication forks.

## Introduction

The proliferating cell nuclear antigen (PCNA) has central roles in DNA replication and repair processes [1]. In its homo-trimeric, ring-shaped configuration, the PCNA clamp tethers DNA polymerase δ and ε, and slides along DNA to promote processive DNA replication. In addition to docking core DNA replication factors, PCNA is also a recruiting platform for components of various DNA repair pathways, including those that drive translesion bypass. Consistent with roles in DNA repair processes, hypomorphic mutation of PCNA has been associated with clinical manifestations of human DNA repair disorders, including neurodegeneration, short status and photosensitivity [2].

The functionality of PCNA entails coordinated opening of its ring structure, its loading onto DNA, and the subsequent ATP hydrolysis-driven closing of the clamp [3]. Although the molecular basis for the assembly of the sliding clamp onto DNA is established [4, 5], mechanisms that detail how PCNA is unloaded have only recently emerged. Indeed, the ATAD5/Elg1-replication factor C-like complex, which has long been known to interact with the replication clamp, was only recently implicated a PCNA-unloading function, although its biochemistry remains undefined [6–8]. Importantly, PCNA residency at replication forks correlated with histone supply [9], and is negatively regulated by cell exposure to replication inhibitors hydroxyurea (HU) and aphidicolin [9–13]. In support of coordinated unloading of PCNA during cell responses to replicative stress, the DNA damage kinases ATM/TEL1 and ATR/MEC1 have documented roles in active unloading of the clamp from damaged forks [14]. Although it remains obscure how PCNA is dynamically regulated, genetic mutations that compromise its homeostasis at stress forks are closely linked to defects in replication restart and checkpoint control [15, 16].

We arrived at TRAIP (aka. RNF206), a RING domain-containing E3 ubiquitin ligase [17], as a factor that localizes at DNA replication compartments during our search for new players in DNA damage responses (DDRs). Human TRAIP and its *Drosophila* homolog NOPO are essential for cell proliferation and organismal development [18–20]. Although TRAIP was originally identified as a negative regulator of NF-κB activation [21–24], the observations that *nopo* embryos displayed spindle defects and arrested in mitosis lend credence to the idea that NOPO/TRAIP may be important in genome stability maintenance [19]. This is further supported by the fact that inactivation of the DNA damage checkpoint kinase CHK2 alleviated the *nopo*-associated phenotypes [19], suggesting that *nopo* cells may have prematurely entered mitosis with under-replicated DNA [25]. Consistent with putative roles in replicative stress responses, TRAIP was recently identified in a proteome-wide study as a factor that concentrated at stalled forks [26], and has been reported to interact with Y-family translesion DNA polymerases, and via undefined mechanisms, promoted recruitment of polymerase eta onto DNA lesions [27].

## Results

### TRAIP protects genome integrity

**Figure 1:**
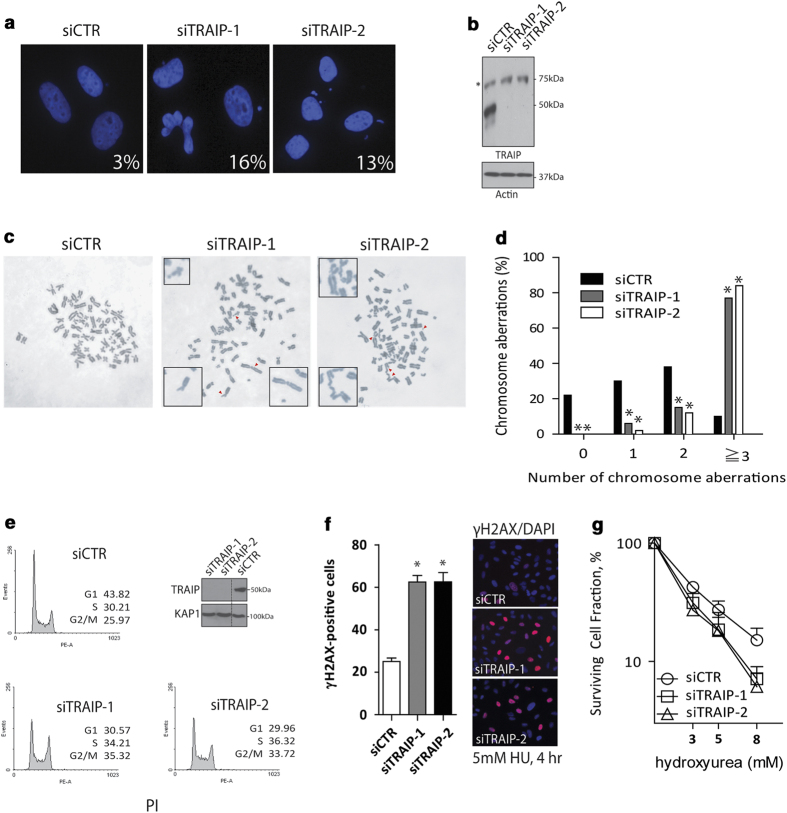
TRAIP protects genome integrity. (**a**–**d**) Cells pre-treated with TRAIP-targeting siRNAs (siTRAIP-1 and siTRAIP-2) or non-targeting control siRNAs (siCTR) were challenged with hydroxyurea (5 mm, 4 h) and were subsequently processed for analysis of micronuclei formation frequencies (**a**), by western blotting to examine knockdown efficiencies (**b**), and frequency of chromosome aberrations (**c**, **d**). For each treatment group, we counted a total of 200 nuclei (**a**) or >100 metaphase spreads (**c**, **d**) from three independent experiments. We scored chromosome breaks and fusions as aberrations (**c**). *P<0.05 vs control. (**e**) Cell-cycle distribution was determined in U2OS cells depleted of TRAIP. Cells pre-treated with indicated siRNAs were fixed in 70% ethanol, stained with propidium iodide (PI), and were subjected to flow-cytometric analysis. (**f**) γH2AX positivity was determined in HU-challenged control or TRAIP-depleted cells by indirect immunofluorescence staining experiments using rabbit polyclonal anti-γH2AX antibodies. We scored cells positive for γH2AX when γH2AX foci >20 per nucleus. Results (mean±s.e.m.) were from three independent experiments with cell count *n*>200 each. *P<0.05 vs control. (**g**) Control or TRAIP-depleted cells were subjected to different doses of HU treatment in the clonogenic survival assay. Cells seeded on 60 mm dishes were incubated at indicated doses of HU-containing medium for 8 h. Results (mean±s.e.m.) were from three independent experiments each performed in triplicates.

With an interest in exploring the putative roles of TRAIP in genome integrity protection, we first examined whether TRAIP inactivation may affect genome stability. Consistent with a recent report [28], analysis of time-lapse fluorescence microscopy images of GFP-H2B-expressing HeLa cells revealed an important role of TRAIP in promoting ordered chromosome segregation, as TRAIP-depleted cells displayed a marked increase in the number of mitotic aberrations, including misaligned and lagging chromosomes (Supplementary Figure S1A and B). Although inactivation of TRAIP using two independent small-interfering RNAs (siRNAs) did not noticeably affect duration of and cell percentage in mitosis (Supplementary Figure S1C and D), TRAIP depletion resulted in a substantial increase in the formation of micronuclei (Figure 1a and b) and gross chromosomal aberrations (Figure 1c and d). Flow-cytometric analysis of cell-cycle profiles also revealed that the majority of TRAIP-depleted cells accumulated in S and G2 phases of the cell cycle (Figure 1e), suggesting that TRAIP may preserve genome integrity during DNA replication [19, 27]. Finally, we found that TRAIP-depleted cells were comparably hyper-responsive to HU treatment, as determined by cell labeling using anti-γH2AX antibodies (Figure 1f) and by clonogenic survival assay (Figure 1g). Together, these observations support the idea that TRAIP is a component of the mammalian DDR network and is important for maintenance of genome stability.

### TRAIP resides in the nucleoli

**Figure 2:**
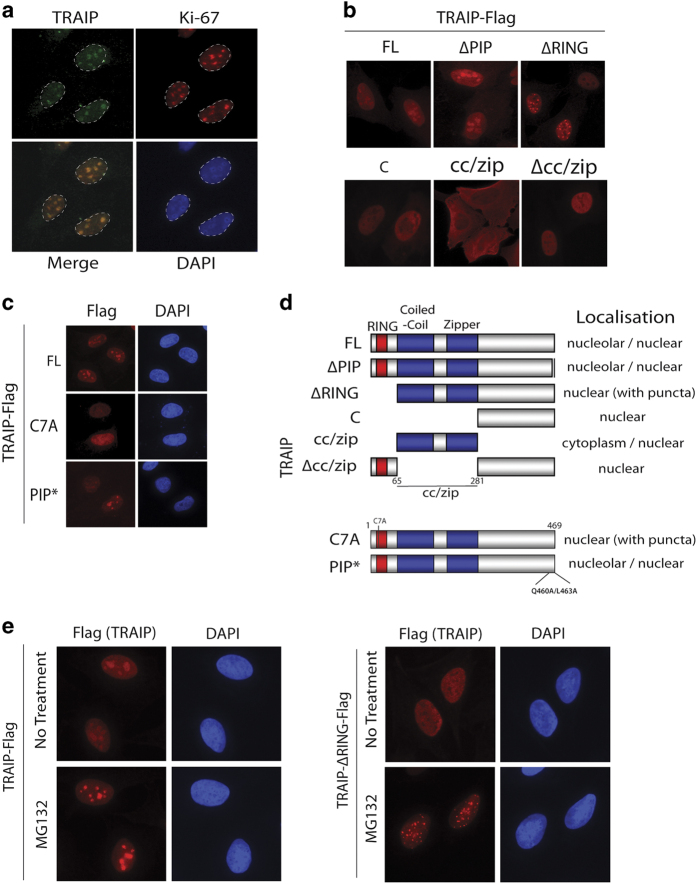
TRAIP resides in the nucleolus. (**a**) TRAIP localizes predominantly in the nucleoli. U2OS cells grown on coverslip were fixed with 3% PFA, permeabilized in 0.5% Triton X-100, and immuno-labeled for TRAIP and Ki-67. Nuclei were visualized by 4,6-diamidino-2-phenylindole (DAPI). (**b**–**d**) Subcellular localization of TRAIP and its mutants. U2OS cells grown on coverslips were transiently transfected with indicated TRAIP cDNAs and thereafter processed for immunofluorescence staining experiments using anti-Flag (M2) antibodies. Schematic illustration of TRAIP full-length (FL) and mutants and a summary of their localization are shown (**d**). ΔPIP has an 8 amino acid internal deletion (amino acids 460–466), whereas C7A and PIP* encode a Cys to Ala and a Gln 460 Ala/Leu 463 Ala mutant, respectively. (**e**) Cells expressing TRAIP-Flag (or its RING deletion mutant; ∆RING) were processed for indirect immunofluorescence experiments following treatment with MG132 (10 μm, 4 h) as described for (**b**). Nuclei were stained with DAPI.

Aside the possibility of residing in the cytoplasm [21–23], TRAIP has also been reported to concentrate in discrete nuclei foci [19, 27–31]. Because of the obscurity of its subcellular localization, we first expressed TRAIP as Flag-epitope fusion proteins to determine where ectopically expressed TRAIP localizes. Interestingly, when the Flag epitope tag was fused to its N-terminus, TRAIP concentrated into nuclear punctate structures similar to those originally described by Lee and colleagues [19]. Strikingly, when epitope-tagged at its C-terminus, TRAIP predominantly concentrated in nucleoli (Supplementary Figure S2A). In support of its nucleolar localization, using rabbit polyclonal antibodies that were raised against TRAIP proteins, we found that endogenous TRAIP also resided in nucleoli (Figure 2a and Supplementary Figure S2B) [27–29]. To further understand the determinants that promote TRAIP occupancy in nucleoli, we generated a number of deletion mutants on the TRAIP polypeptide and assessed their subcellular localization (Figure 2b–d). Intriguingly, we found that TRAIP RING mutants (ΔRING and C7A) did not accumulate in nucleoli, but instead formed speckle-like structures that are most prominent when the mutant proteins are expressed at high levels. Indeed, proteasomal inhibition increased the expression and the formation of TRAIP ΔRING speckles, but not that of wild-type TRAIP (Figure 2e). Together, we concluded that TRAIP localizes in nucleoli, and that the N terminus of TRAIP may be important in regulating its nucleolar retention.

### TRAIP is a PCNA-interacting factor

**Figure 3:**
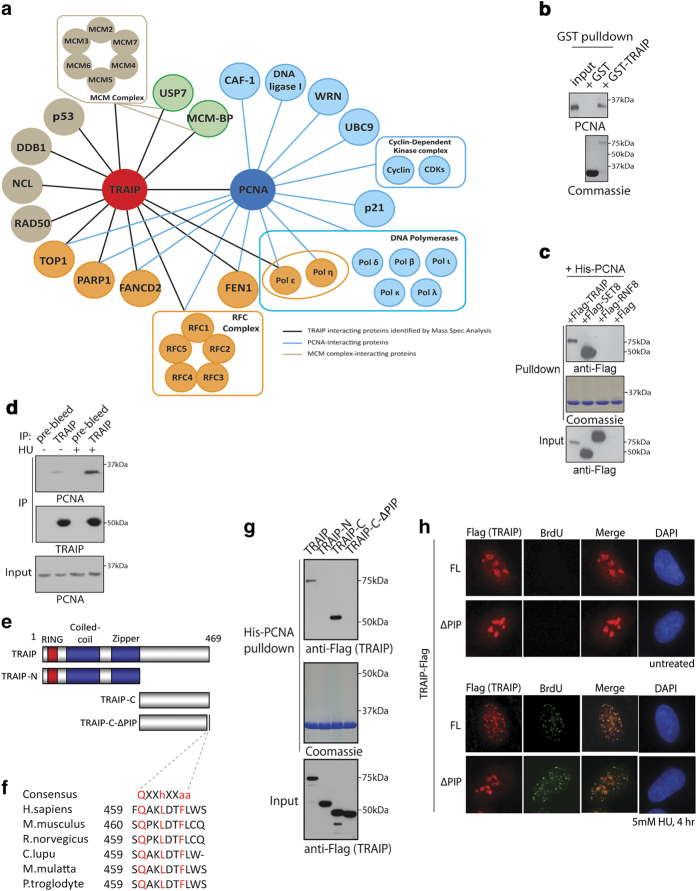
TRAIP is a PCNA-interacting protein. (**a**) Illustration depicting the TRAIP interactome (identified from this study) is shown. TRAIP protein complexes were affinity purified and identities of TRAIP-co-purifying proteins were analyzed by mass spectrometric analysis (LC/MS/MS). Relationships of PCNA and its established interacting partners are also displayed. (**b, c**) TRAIP interacts with PCNA. Bacterially expressed and purified GST or GST-TRAIP fusion proteins were conjugated onto affinity matrix and incubated with 293T cell lysate. The GST–TRAIP–PCNA interaction was examined by western blotting using indicated antibodies (**b**). His-tagged PCNA conjugated onto nickel agarose beads was incubated with lysates derived from cells expressing Flag-epitope tagged TRAIP, SET8 or RNF8. Bound proteins were analyzed by western blotting experiments (**c**). SET8 is a known PCNA-interacting protein and was used as a positive control. Note that expression constructs encoding Flag-TRAIP, SET8 and RNF8 are fused with affinity tags (Streptavidin binding peptide and S-protein tag; see Materials and methods) and are of much large size. (**d**) Replicative stress enhanced the TRAIP–PCNA interaction. 293T cells pre-treated with hydroxyurea (HU) (5 mm, 4 h) were lysed and co-immunoprecipitation experiments were used to examine the TRAIP–PCNA interaction. Western blotting experiments were performed using indicated antibodies. (**e**) Domain organization of TRAIP and its mutants. (**f**) Identification and conservation of the PIP box on TRAIP. TRAIP-C-ΔPIP is an internal deletion mutant and has its conserved PIP box (QAKLDTFLWS) removed. (**g**) TRAIP interacts with PCNA via its PIP. Bacterially expressed His-PCNA proteins were incubated with lysates derived from cells expressing TRAIP and its various mutants (see Figure 2e). (**h**) TRAIP migrates to HU-induced DNA lesions. Cells expressing TRAIP-Flag (or its PIP deletion mutant; ∆PIP) pre-labeled with 10 μM BrdU for 24 h were treated with either HU or not, and were subsequently processed for indirect immuno-fluorescence experiments using anti-Flag (M2) and anti-BrdU (Rat) antibodies. Nuclei were stained with DAPI.

To explore how TRAIP protects genome integrity and to define the TRAIP interactome in response to replicative stress, we affinity-purified TRAIP protein complexes from 293T cells that have been engineered to stably express streptavidin-binding peptide- and S-tagged TRAIP. Mass spectrometric analysis of TRAIP immunoprecipitates identified a cohort of DNA replication and repair factors, including PCNA (Figure 3a). Consistently, reciprocal pull-down experiments confirmed that TRAIP specifically interacted with PCNA (Figure 3b and c), and that pre-treating cells with HU further enhanced the TRAIP–PCNA interaction (Figure 3d).

In line with the observation that many PCNA-interacting proteins bear PIP boxes via which they bind to the DNA replication processivity factor, visual inspection of the TRAIP polypeptide sequence led to the identification of an evolutionarily conserved PIP-like sequence on TRAIP C-terminus (Figure 3e and f). Consistent with a requirement of TRAIP PIP in mediating its PCNA interaction, deletion of the putative PIP on TRAIP impaired its interaction with PCNA (Figure 3g). Because TRAIP normally resides in nucleoli we speculated that TRAIP may migrate out of the nucleoli in response to replicative stress. This would explain how HU potentiated the TRAIP–PCNA interaction (Figure 3d). To test this possibility, we pre-incubated cells with BrdU to facilitate identification of single-stranded DNA (ssDNA) regions that arise during replicative stress [32]. We performed BrdU labeling under native conditions, and determined subcellular localization of TRAIP in HU-challenged cells. Consistently, upon treatment with HU, which slows down DNA synthesis and uncouples it from the helicase activity that unwinds double-strand DNAs ahead of the replication forks, TRAIP accumulated into punctate structures that overlapped with ssDNAs at stalled forks (Figure 3h). Translocation of TRAIP onto HU-induced ssDNA stretches required its PIP box (Figure 3h), resembling its pivotal role in supporting TRAIP accumulation at laser-induced DNA damage tracks (Supplementary Figure S3A–C). TRAIP also relocalized to γH2AX-marked sites in response to other types of replicative stress-inducing agents, including aphidicolin, mitomycin C, UV radiation and cisplatin (Supplementary Figure S3D). By contrast, TRAIP did not noticeably accumulate at IR-induced DNA lesions. Together, these data implicate a direct role of TRAIP, via its PIP, at stressed replication forks.

### TRAIP inactivation compromised replication processivity

**Figure 4:**
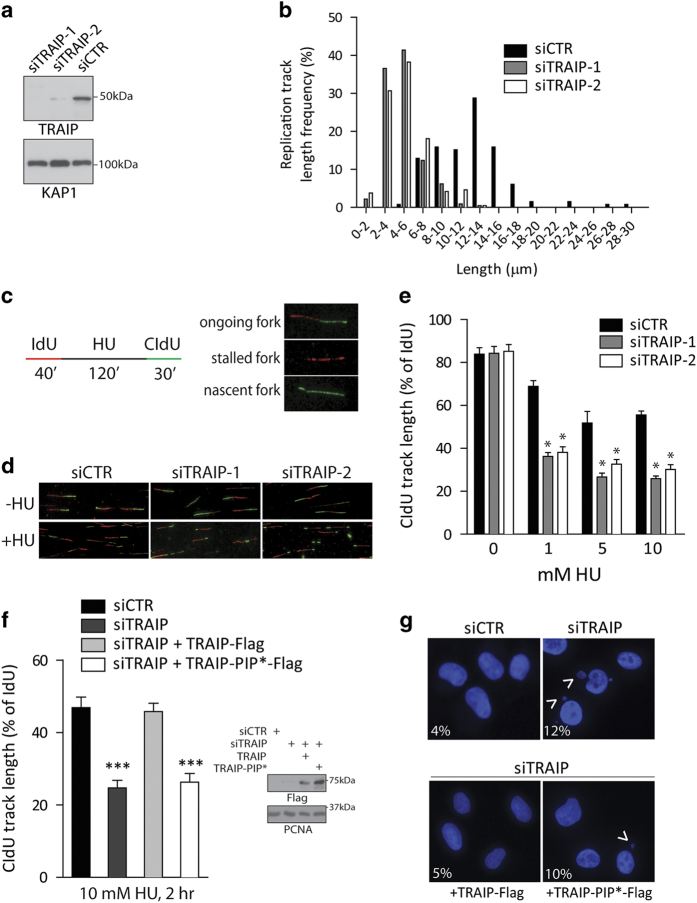
TRAIP inactivation compromises DNA synthesis during replicative stress. (**a**, **b**) TRAIP-depleted cells displayed reduced rates of fork progression under replicative stress. Cells pre-treated with TRAIP-targeting siRNAs (siTRAIP-1 and siTRAIP-2) or control siRNAs (siCTR) were sequentially labeled with IdU then with CIdU in the presence of 5 mm hydroxyurea (HU). Western blotting experiments were performed to assess TRAIP silencing efficiency using anti-TRAIP antibodies. KAP1 was used as a loading control (**a**). DNA combing assays were performed to examine DNA synthesis, and length of CIdU tracks was determined (**b**) from three independent experiments. Note that IdU tracks did not differ among the treatment groups. (**c–e**) TRAIP promotes recovery of stalled forks. Schematic depicting experimental procedures involving sequential labeling with IdU and CIdU (**c**). DNA from cells pre-treated with TRAIP-specific siRNAs (siTRAIP-1 and siTRAIP-2) or control siRNAs (siCTR) was combed and immuno-labeled to determine fork progression with (+HU; 10 mm, 2 h) or without HU (−HU) treatment (**d**, **e**). Quantification of CIdU-labeled tracks relative to IdU tracks is shown for a range of HU doses. *P<0.05 vs control. (**f**) TRAIP promotes DNA replication restart via its PIP. Cells expressing siRNA-resistant TRAIP cDNAs (TRAIP-Flag or its PIP* mutant) were transfected with indicated siRNAs. As depicted in (**c**) cells were labeled with IdU, challenged with 10 mm HU (2 h), were labeled with CIdU before they were lysed and processed to assay for DNA synthesis. ***P<0.05 vs control. Western blot showing expression of TRAIP-Flag and mutant is shown. (**g**) Images showing micronuclei formation (arrow heads) in HU-challenged (5 mm, 4 h) cells pre-treated with control siRNA (siCTR), TRAIP-targeting siRNAs (siTRAIP), or in cells reconstituted with siRNA-resistant TRAIP cDNAs as in (**f**). Frequencies of micronuclei formation are shown. Results were derived from three individual experiments (*n*=200).

In light of the stress-inducible relocalization of TRAIP onto stalled forks and its interaction with the PCNA clamp, we speculated that TRAIP may be important in repair of damaged forks. To this end, we inactivated TRAIP using the RNA interference approach, and analyzed fork progression by use of the DNA combing method. Accordingly, we sequentially labeled cells with thymidine analogs (IdU and CIdU) in control and TRAIP-knockdown cells to compare relative rates of DNA replication in the absence or presence of HU. Interestingly, although TRAIP deficiency did not noticeably affect DNA synthesis in unperturbed cells, length of CIdU tracks, reflective of DNA synthesis under stress, was substantially shorter in TRAIP-depleted cells (Figure 4a and b), suggesting that TRAIP deficiency compromises fork stability and progression when nucleotide pool becomes limiting.

We also examined whether TRAIP may be required for fork restart using the same methodology (Figure 4c). Cells were first labeled with IdU to control for rates of unperturbed DNA synthesis. Following a 2-h hydroxyurea challenge, cells were incubated with CIdU to assay fork restart and progression. Notably, depletion of TRAIP resulted in much shorter CIdU tracks over a wide dose range of hydroxyurea treatment (Figure 4d and e), suggesting that TRAIP inactivation hampered fork restart or stability. Our observation that most forks restarted upon HU withdrawal precludes complication that may arise from fork collapsing (Supplementary Figure S4A-C), which results only after prolonged HU treatment [33]. Indeed, prolonged inhibition of *de novo* nucleotide synthesis compromised fork restart irrespective of TRAIP status (Supplementary Figure S4D–G).

To examine the functional relevance of the TRAIP–PCNA interaction in promoting recovery of stalled forks, we generated siRNA-resistant versions of TRAIP (Supplementary Figure S5), and performed reconstitution experiments in TRAIP-depleted cells. Notably, in contrast to their wild-type counterparts, cells expressing the PCNA-binding defective TRAIP mutant (TRAIP-PIP*) displayed defects in fork progression during cell recovery from HU challenge (Figure 4f), and failed to alleviate the TRAIP deficiency-associated increase in micronuclei formation (Figure 4g). Taken together, these data highlight the TRAIP–PCNA axis in repair and recovery of HU-stalled forks.

### TRAIP regulates PCNA level on chromatin

**Figure 5:**
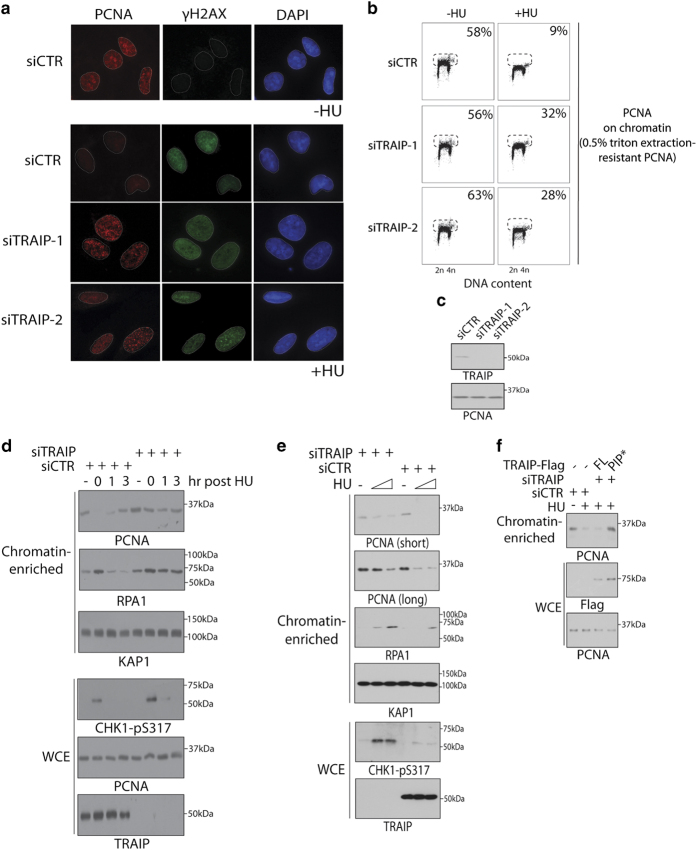
TRAIP promotes PCNA unloading in response to replication inhibition. (**a**) U2OS cells transfected with indicated siRNAs were grown on coverslips, and were either incubated with 5 mm hydroxyurea (+HU) or left untreated (−HU) for 4 h. Cells were subsequently processed for indirect immunofluorescence experiments using anti-PCNA and anti-γH2AX antibodies. Nuclei were visualized by DAPI staining. (**b**) U2OS cells transfected with indicated siRNAs were either incubated with 5 mm HU (+HU) or left untreated (−HU) for 1 h. Cells were trypsinized, permeabilized in 0.5% Triton X-100 for 10 s and thereafter fixed in methanol. To detect chromatin-bound PCNA, cells were labeled with anti-PCNA antibodies. Processed cells were analyzed by flow cytometry, and cells positive for PCNA are shown in percentages. (**c**) Representative blot showing siRNA-mediated TRAIP knockdown in U2OS cells. (**d**) U2OS cells pre-treated with TRAIP-targeting siRNAs were incubated with HU (10 mm, 2 h) and released. Cells were harvested at indicated time points, and were biochemically fractionated to determine PCNA level on chromatin-enriched fractions. (**e**) U2OS cells were transfected with indicated siRNAs and were incubated with 0, 2 or 10 mm HU for 2 h. Cells were processed as in (**d**). (**f**) U2OS cells or derivatives that express siRNA-resistant versions of TRAIP (full-length, FL or PIP*) cDNAs were transfected twice with TRAIP-targeting siRNAs (siTRAIP-1) or control (siCTR) at 24 h intervals. Forty-eight hours post transfection, cells were challenged with 10 mm HU for 2 h. Whole cell extract (WCE) and chromatin-enriched fractions were prepared, and were processed for western blotting analysis using indicated antibodies.

Replication inhibition triggers unloading of PCNA from DNA replication factories [9–13]. Given the importance of the TRAIP-PCNA interaction in replicative stress responses, we explored whether TRAIP may regulate chromatin binding of PCNA following HU treatment. Noting that replication fork-associated PCNA proteins at DNA replication factories can be visualized by incorporating a pre-extraction step before cell fixation, we pre-extracted the soluble pool of PCNA using 0.5% Triton X-100 solution before cells were fixed in methanol. Intriguingly, in sharp contrast to control cells, where intensity of PCNA accumulation at DNA replication factories was markedly reduced when cells were challenged with HU (Figure 5a and Supplementary Figure S6), this was not seen in cells depleted of TRAIP (Figure 5a). Consistently, flow-cytometric analysis of the triton extraction-resistant pool of PCNA also revealed an important role of TRAIP in HU-induced PCNA unloading (Figure 5b and c), suggesting that TRAIP may participate in regulating PCNA unloading from chromatin during replicative stress.

Similar to yeast cells [14], HU-induced PCNA unloading from chromatin required the DNA damage kinases ATM and ATR (Supplementary Figure S7A–E), although chemical inhibition of ATM/ATR did not compromise TRAIP relocalization onto ssDNAs (Supplementary Figure S7F). To further explore the possibility of a role of TRAIP in promoting PCNA unloading in response to replicative stress, we biochemically fractionated chromatin-enriched fractions from control and stressed cells, and assessed the level of PCNA immediately after and following recovery from HU treatment. Importantly, we found reproducibly that chromatin-bound PCNA level was much reduced in response to HU, and that it gradually recovered upon HU withdrawal (Figure 5d). By contrast, TRAIP inactivation impaired the HU-induced reduction of PCNA on chromatin, and was coupled with hyper-accumulation of ssDNA-binding protein RPA. Hyper-accumulation of RPA was also observed in TRAIP-depleted cells in response to a range of replicative stress-inducing agents (Supplementary Figure S8). Interestingly, the effect of chemical inhibition of *de novo* nucleotide synthesis on PCNA unloading from chromatin was dose dependent, and was inversely proportional to RPA loading (Figure 5e). It is noteworthy to mention that the PCNA and RPA dynamics at stressed forks observed in these experimentations share striking similarities to those observed previously in live cells at single-cell level [10]. Given the requirement of TRAIP PIP in its PCNA targeting, we performed reconstitution experiments in TRAIP-knockdown cells to assay the functional relevance of the TRAIP–PCNA interaction. Using biochemical fractionation approach to monitor chromatin-bound PCNA, we found that HU-regulated PCNA unloading also required the TRAIP PIP box (Figure 5f), highlighting a requirement for PCNA targeting in this process *in vivo*. Together, these data implicate TRAIP in regulating stress-induced PCNA turnover at replication forks.

Our observation that replicative stress triggered substantial reduction of PCNA from stalled forks, together with the prior knowledge that TRAIP encodes an active E3 ubiquitin ligase prompted us to speculate whether TRAIP may ubiquitylate and promote PCNA turnover. Given that mutations of TRAIP RING perturbed its subcellular localization, which precluded us from directly examining its functionality *in vivo*, we chemically inhibited the proteasome using MG132 and assayed for recovery of stalled forks in cells. We reasoned that whether TRAIP promotes PCNA ubiquitylation and turnover, proteasome inhibition should mimic TRAIP inactivation, and should exacerbate the HU-induced defects in fork progression and recovery. Despite established roles of the ubiquitin proteasome pathways in cell-cycle control regulation, transient inhibition of the proteasome did not exacerbate HU-induced fork stalling (Supplementary Figure S9), although it did reveal that TRAIP expression is kept at low levels in cells (Supplementary Figure S10). Failure to limit TRAIP activity, perhaps by restricting its nucleolar residency, may trigger unscheduled PCNA unloading. Interestingly, cells seem to have evolved strategies to sequester unrestrained TRAIP proteins into nuclear bodies that do not overlap with known entities (Figure 2 and Supplementary Figure S2A) [19].

## Discussion

Serving a master scaffolding role at DNA replication forks the PCNA clamp is dynamically regulated to coordinate processive DNA replication with translesion synthesis and checkpoint activation. In this study, we have identified TRAIP as a novel PCNA-interacting factor that promotes recovery of damaged forks. TRAIP bears a PIP-like motif at its C-terminus (Figure 3e and f), relocalizes to stress-induced ssDNAs (Figure 3h) and γH2AX-marked DNA lesions (Supplementary Figure S3D), and targets PCNA to facilitate its turnover from chromatin (Figure 5). Consistent with a direct role at damaged forks, TRAIP inactivation resulted in severely compromised DNA synthesis during nucleotide scarcity (Figure 4), which in turn jeopardized genome stability (Figure 1 and Supplementary Figure S1). Together, our findings uncover TRAIP as a component of the mammalian replicative stress response network, and implicate dynamic PCNA turnover as a TRAIP-dependent mechanism in promoting faithful duplication of the genetic material.

**Figure 6:**
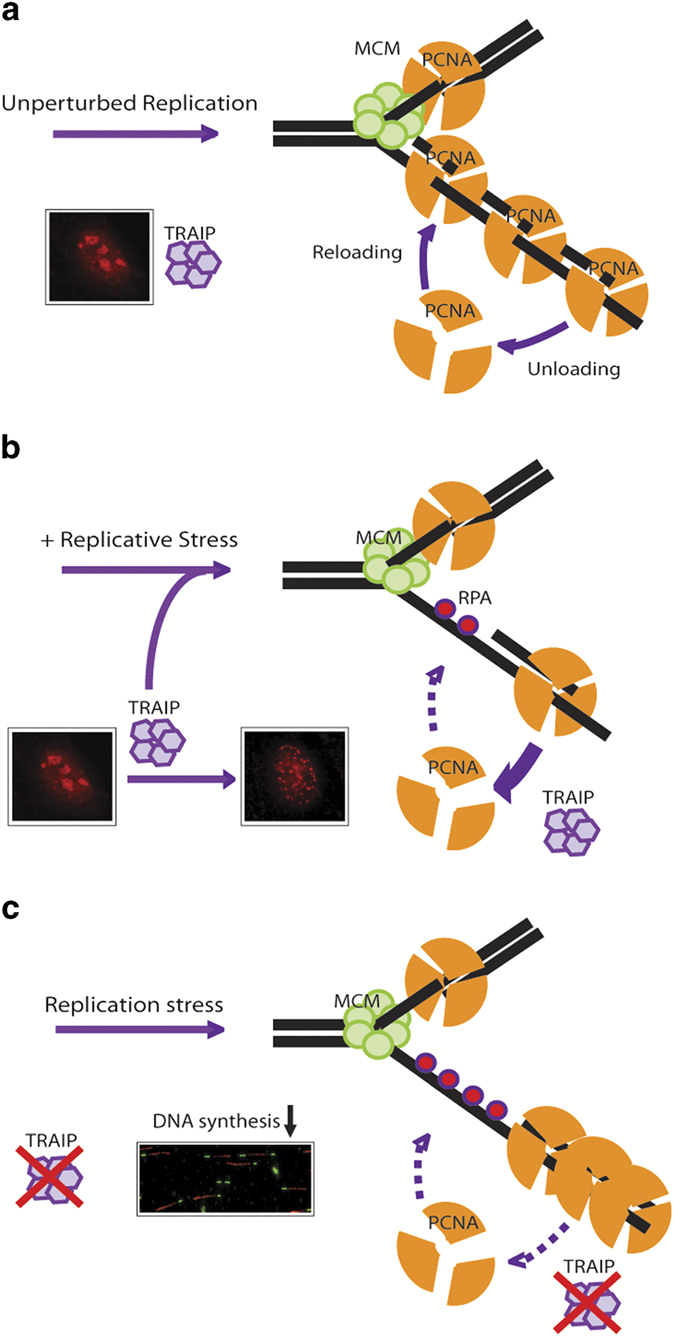
Working model depicting how TRAIP participates in regulating PCNA unloading from stressed replication forks. PCNA is dynamically loaded and unloaded at replication forks in proliferating cells (**a**). TRAIP senses nucleotide shortage and translocates to stressed replication forks where it promotes PCNA unloading via an undefined mechanism (**b**). In the absence of TRAIP, PCNA turnover from chromatin is perturbed and DNA synthesis is compromised (**c**).

The nucleolus serves as a reservoir for many DDR proteins, including the Werner’s syndrome gene product [34]. Interestingly, our findings that TRAIP proteins concentrate in nucleoli in a RING-dependent manner raise the exciting possibility that the E3 ubiquitin ligase signature motif, rather than serving catalytic roles, may provide a structural scaffold to promote TRAIP occupancy in these subcellular compartments (Figure 2b and e). This notion is further corroborated by the fact that N-terminally tagged TRAIP fusion proteins, in sharp contrast to its C-terminally tagged counterparts, are mislocalized (Supplementary Figure S2A). Although it has been reported to enhance ubiquitylation of a translesion polymerase [27], our findings that TRAIP RING is required for its proper subcellular localization precluded us from testing directly whether its E3 ubiquitin ligase activity may be important for its functionality in recovery of damaged forks, as RING-inactivated TRAIP mutants have lost their inducibility to translocate from nucleoli to stressed forks, and is further complicated by their tendency to form nuclear punctae of unknown nature (Figure 2b and e). Following this argument, while we note that the TRAIP deficiency-associated increase in micronuclei formation frequency was suppressed by the re-introduction of wild-type TRAIP, but not its RING-inactivated mutant (Supplementary Figure S11A), further experimentations will be necessary to ascribe a direct role of TRAIP RING in replicative stress responses. As TRAIP inactivation did not noticeably affect the level of ubiquitylated PCNA in response to replicative stress (Supplementary Figure S11B), and that proteasome inhibition did not compromise fork recovery upon relief of replicative stress conditions (Supplementary Figure S9), we favor the model in which TRAIP may promote PCNA unloading in a non-catalytic manner (Figure 6). Notably, the idea that E3 ubiquitin ligases also serve non-catalytic roles is not unprecedented among enzymes in the RING family [26, 35, 36]. Anyhow, given that emerging link between replicative stress and fork dissociation of PCNA [9–13, 37], it will be of significant interest to define mechanistically how TRAIP participates in this process.

Our analysis of the TRAIP interactome highlighted important roles of TRAIP in DNA replication processes. Indeed, not only does TRAIP interact with the core DNA replication factor PCNA (Figure 3), inactivation of TRAIP, or its interaction with PCNA, compromised DNA synthesis at times of nucleotide shortage (Figure 4). It is noteworthy to mention, however, that our identification of the TRAIP–PCNA axis in replicative stress responses does not exclude the possibility that TRAIP may also participate in driving ordered cell progression through mitosis as reported recently by the Huber and Kim laboratories, despite of the discrepancies of the two studies [28, 38]. Notably, our finding that mitotic indices of TRAIP-depleted cell populations did not noticeably differ from control cells is in general agreement with those described by Huber and co-workers, although the authors also proposed a positive role of TRAIP in the spindle assembly checkpoint [28].

To ensure faithful duplication of its genetic material, cells have evolved various DNA repair and tolerance pathways to accommodate many different types of DNA damage during DNA replication. As such, one could envisage that nucleotide shortage and damaged DNA templates may trigger very different responses at replication forks. Indeed, it has become clear that chemical inhibition of *de novo* nucleotide synthesis leads to active PCNA unloading from replication compartments [9–12], whereas similar observation is not seen in cells in response to UV irradiation. Importantly, and as pointed out by Dobrucki and colleagues [37], given the established roles of PCNA in DNA repair, the differences in PCNA foci dynamics could be a result of the need to mobilize and reload PCNA for repair of certain types of DNA lesions, and warrants a more in-depth study of TRAIP and PCNA dynamics at DNA replication factories. Nevertheless, our findings that TRAIP relocalizes in response to a range of genotoxic agents clearly suggest broader roles of the E3 ubiquitin ligase in the mammalian cell responses to replicative stress (Supplementary Figure S3D).

Although PCNA unloading is clearly an important molecular event at stressed replication forks, it remains obscure why PCNA clamps need to be cleared from the scene. One possibility is that defective unloading of PCNA impairs its recycling, limits its availability and in turn slows down fork progression. Alternatively, one may speculate that stress-induced PCNA unloading may be coupled to fork remodeling [16], which in turn may facilitate replication fork reversal and restart [39, 40]. This model would predict that failure to efficiently unload PCNA from stressed replication forks compromises fork remodeling, and would negatively regulate restart of DNA replication. Problematic fork recovery can also contribute to under-replicated DNA and consequently genome instability. Although this model remains to be definitively tested, our data clearly showed that TRAIP inactivation hampered fork restart and progression during and upon recovery from HU challenge (Figure 4), and that TRAIP depletion resulted in hyper-accumulation of ssDNA-binding protein RPA on chromatin (Figure 5d and e, Supplementary Figure S8). Given the intimate links of PCNA and DNA repair, and the TRAIP-PCNA interaction in the dynamic regulation of PCNA on chromatin, it would be of significant interest to explore how TRAIP mutations may contribute to clinical manifestations of human DNA repair disorder, including the recently described primordial dwarfism [41].

## Materials and methods

### Antibodies and chemicals

The TRAIP polyclonal antibody was raised against GST-TRAIP-N terminal fusion protein (see Figure 3e) and affinity purified using column coated with MBP-TRAIP-N terminal fusion protein. Antibody specifically recognizing γH2AX was previously described [42]. The anti-PCNA (PC10) and anti-CHK1 (G-4) antibodies were from Santa Cruz (Dallas, TX, USA); anti-Ki67 antibodies were from Chemicon (Darmstadt, Germany); anti-RPA1 antibodies were from Calbiochem (Darmstadt, Germany); anti-Chk1-pS345 antibodies were from Cell Signaling (Danvers, MA, USA); anti-KAP1 antibodies were from BD Transduction Laboratories (San Jose, CA, USA). Anti-Actin, anti-GFP and anti-Flag (M2) antibodies were obtained from Sigma (Darmstadt, Germany). ATMi (KU55993) and ATRi (VE821) inhibitors were from SelleckChem (Houston, TX, USA).

### Tandem affinity purification

Affinity purification of TRAIP protein complexes was carried out essentially as described previously [43]. Briefly, 293T cells expressing TRAIP with C-terminally tagged with streptavidin binding peptide-S peptide-Flag (SFB) were treated with 5 mm HU for 4 h, harvested and lysed in NETN buffer (100 mm NaCl, 1 mm EDTA, 20 mm Tris-HCl pH 8 and 0.5% Nonidet P-40) for 15 min on ice. Supernatant containing TRAIP–protein complexes was purified by sequential binding to streptavidin sepharose (Amersham Bioscience, Pittsburgh, PA, USA) and S protein agarose (Novagen, Darmstadt, Germany), and was subjected to mass spectrometric analysis (LC/MS/MS) at Taplin Mass Spectrometry Facility (Harvard).

### Cell culture and transfection

U2OS, HeLa and 293T cells (or their derivatives) were cultured in DMEM supplemented with 10% fetal calf serum and maintained in 5% CO_2_ at 37 °C. Cell transfection was performed using Polyethylenimine (PEI; Polysciences Inc., Warringston, PA, USA).

### Small-interfering RNAs

siRNAs targeting TRAIP or non-targeting control siRNAs (Dharmacon, Lafayette, CO, USA) were transfected twice at 24-h intervals using Oligofectamine (Invitrogen, Carlsbad, CA, USA). The TRAIP siRNA sequences are CAG ACA GUC UAC UCU GAA UTT (siTRAIP-1) and CAG CAU GGU UAC UAC GAA ATT (siTRAIP-2). siRNAs were used at a final concentration of 100 nm.

### Protein–protein interacting studies

To examine the interaction between TRAIP and PCNA, GST-TRAIP and GST were expressed and purified from BL21 bacterial cells using standard procedures, conjugated onto GST beads, and were incubated with lysates derived from 293T cells at 4 °C for 4 h. Beads were washed twice with NETN buffer. Proteins were eluted by boiling in Laemmli sample buffer, separated by SDS-PAGE, and immunoblotting experiments were performed. For histidine (HIS) tagged-PCNA pulldown experiments, constructs encoding Flag-tagged TRAIP and its mutants were transiently transfected into 293T cells. Cells were lysed in NETN buffer, cleared by centrifugation before they were incubated with purified His-PCNA on nickel beads for 4 h at 4 °C. Beads were subsequently washed, boiled in Laemmli sample buffer, separated by SDS-PAGE and immunoblotted with the indicated antibodies. To detect the endogenous TRAIP-PCNA complex, 293T cells pre-treated with or without HU (5 mm, 4 h) were lysed, and immunoprecipitation was performed using anti-TRAIP sera or pre-bleed control.

### Immunostaining procedure

Unless otherwise stated, cells grown as monolayers on coverslips were permeabilized in 0.5% Triton X-100 solution for 10 s, following by fixation in 3% paraformaldehyde at room temperature for 15 min. Nuclei were visualized by staining with DAPI. Images were acquired using an Olympus BX51 fluorescence microscope (Tokyo, Japan). To visualize PCNA replication foci or for examination of chromatin-bound PCNA by flow cytometry, cells were pre-extracted with 0.5% Triton X-100 solution for 10 s followed by a 20-min methanol fixation step at −20 °C.

### DNA fiber assays

U2OS cells were sequentially labeled with IdU (50 μm) and CidU (100 μm). Thereafter, labeled cells were harvested and DNA fiber spreads were prepared on silanized slides (DAKO, Santa Clara, CA, USA) by addition of 200 mm Tris-HCl pH 7.5, 50 mm EDTA, 0.5% SDS [44]. We fixed and denatured DNA fibers using methanol/acetic acid (3:1), and 2.5 m HCl, respectively. The acid-treated fiber spreads were subsequently neutralized with Borax buffer (0.1 m borax, pH 8.5), and co-stained with mouse anti-BrdU antibodies (BD Biosciences, clone B44; diluted at 1:100) and rat anti-BrdU antibodies (AbD Serotec, Hercules, CA, USA, clone BU1/75; diluted at 1:100). Secondary antibodies were Rhodamine goat anti-mouse IgG and FITC goat anti-rat IgG (both from Jackson ImmunoResearch Laboratories, West Groves, PA, USA; diluted at 1:400). Fibers were examined under fluorescence microscope. For quantification of replication structures, at least 250 structures were counted per experiment. The relative lengths of red (Rhodamine) and green (FITC) labeled patches were measured using the ImageJ software (National Institutes of Health; http://rsbweb.nih.gov/ij).

### Laser microirradiation

To generate localized DNA damage, cells were plated on glass-bottomed dishes (World Precision Instruments Inc., Sarasota, FL, USA) and pre-sensitized with 10 μm 5-bromo-2′-deoxyuridine (BrdU; Sigma-Aldrich) for 24 h at 37 °C. We performed laser microirradiation by using an inverted confocal microscope (LSM 510 Meta; Carl Zeiss, Inc., Oberkochen, Germany) equipped with a multiphoton system with single scan iteration at 800 nm, 10% output power of 1 800 mW. Cells were imaged with a ×63 Plan-Apochromat/1.4 NA oil objective.

### Time-lapse microscopy

H2B-GFP expressing HeLa cells were seeded, and were transfected with control or TRAIP siRNAs. Twenty-four hours post transfection cells were placed in a live cell stage-mounted environment chamber and images were captured at 5 min intervals using Perkin Elmer Spinning Confocal Microscope (Waltham, MA, USA) for 8 h. Data were analyzed using the MetaMorph analysis software (Sunnyvale, CA, USA).

### Metaphase spreads

HeLa cells were treated with 0.5 μm nocodazole for 4 h and lysed with 75 mm KCl in 37 °C for 30 min. Cells were fixed on ice with a 3:1 methanol/acetic acid solution. Cell were then dropped onto slides, allowed to dry and were stained with Giemsa Stain (Gibco, Waltham, MA, USA). Numbers of chromosome aberrations per metaphase were counted. We scored chromosome breaks and fusions as chromosome aberrations.

### Cell-cycle analyses

U2OS cells were trypsinized and fixed with 70% ethanol. Cells were washed with PBS, incubated with RNAse for 30 min at 37 °C, and stained with propidium iodide for 10 min. Cell-cycle distribution was determined using a BD FACSCantoII Analyzer (San Jose, CA, USA).

### Protein purification

PCNA was expressed in BL21 cells and purified using metal chelate affinity chromatography via an N-terminal His-tag. Briefly, harvested cell pellet was resuspended in lysis buffer (20 mm Tris-HCl pH 8.0, 300 mm NaCl, 20 mm imidazole, 10% (v/v) glycerol, 1 mm PMSF) and lysed by sonication. After centrifugation, the supernatant was applied to a 1-ml Ni-NTA resin pre-equilibrated with lysis buffer. The resin was washed extensively with lysis buffer, followed by additional 10-column volumes of lysis buffer containing 40 mm imidazole. Proteins were eluted with 300 mm imidazole in lysis buffer. Pooled fractions were buffer-exchanged and concentrated using a 30 K MWCO concentrator (Millipore Corporation, Billerica, MA, USA). Purified proteins were divided into small aliquots, snap-frozen in liquid nitrogen and stored at −80 °C until use.

### Chromatin fractionation

To examine chromatin-bound PCNA, cells were pre-extract with NETN buffer for 15 min on ice (soluble fraction). Thereafter, the pellet was solubilized with NETN buffer supplemented with Turbonuclease (chromatin-enriched fraction).

### Statistical analysis

Where appropriate, Student’s *t*-test was used to evaluate potential differences between control and treatment groups. We reported statistical significance at *P*<0.05.
