# Supplementary material for: TRAIP regulates replication fork recovery and progression via PCNA
Source: Cell Discov. 2016 Jun 28;2:16016–. doi: 10.1038/celldisc.2016.16 (PMC4923944; doi:10.1038/celldisc.2016.16)
Supplement: Supplementary Figure S1 [file celldisc201616-s1.pdf]

Supplementary Figure S1

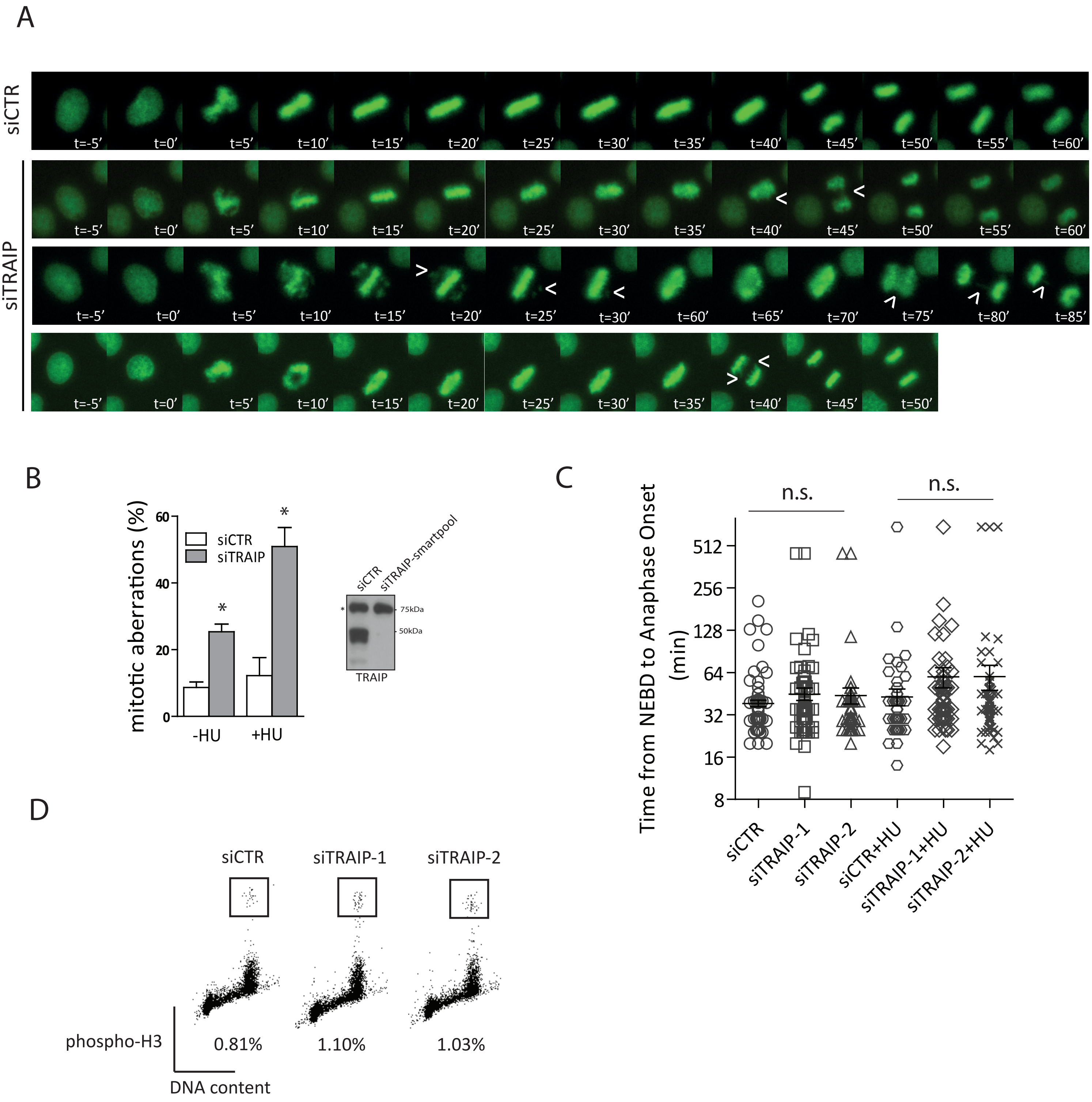

Supplementary Figure S1
A - B) HeLa GFP-H2B expressing cells pre-treated with TRAIP-targeting pooled siRNAs (siTRAIP; Dharmacon SMARTpool) or control siRNAs (siCTR) were incubated with or without hydroxyurea (5mM HU, 4 hr) and were monitored by time-lapse microscopy. Images of HU-challenged control and TRAIP-depleted cells undergoing mitosis are shown (A) and the percentage of cells that displayed aberrant mitotic structures is displayed (B). Results represent mean±S.E.M. from three individual experiments n=20 each. We scored misaligned and lagging chromsomes as mitotic aberrations (arrow heads). *p<0.05 vs. control; C) HeLa GFP-H2B expressing cells pre-treated with indicated siRNAs were monitored by time-lapse microscopy. Elapsed time (min) between nuclear envelope breakdown (NEBD) and anaphase onset for individual cells is plotted (C), n>30; D) Mitotic cell population in TRAIP-inactivated cells. siRNA-treated cells were fixed in 70% ethanol, immuno-stained with anti-H3pS10 antibodies and were subjected to flow cytometry analysis. Percentage of mitotic population is shown. n.s.; not significant.
