# Supplementary material for: TRAIP regulates replication fork recovery and progression via PCNA
Source: Cell Discov. 2016 Jun 28;2:16016–. doi: 10.1038/celldisc.2016.16 (PMC4923944; doi:10.1038/celldisc.2016.16)
Supplement: Supplementary Figure S2 [file celldisc201616-s2.pdf]

Supplementary Figure S2

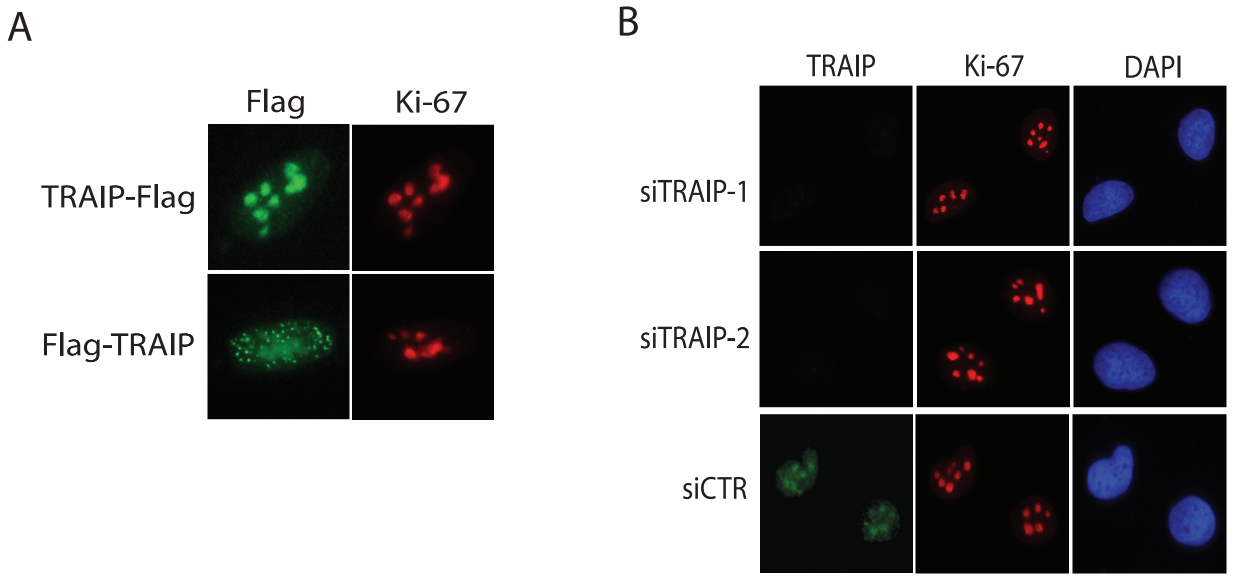

Supplementary Figure S2
A) U2OS cells expressing TRAIP-Flag and Flag-TRAIP were immunostained with anti-Flag (M2) and anti-Ki-67 antibodies. Ki-67 was used as a nucleoli marker; B) U2OS cells transfected with 100 nM of TRAIP siRNAs or a control siRNA (siCTR) were analysed by indirect immunofluorescence studies using anti-TRAIP antibodies. Nucleoli were stained with anti-Ki-67 antibody. Nuclei were counter-stained with 4,6-diamidino-2-phenylindole (DAPI).
