# Supplementary material for: TRAIP regulates replication fork recovery and progression via PCNA
Source: Cell Discov. 2016 Jun 28;2:16016–. doi: 10.1038/celldisc.2016.16 (PMC4923944; doi:10.1038/celldisc.2016.16)
Supplement: Supplementary Figure S3 [file celldisc201616-s3.pdf]

Supplementary Figure S3

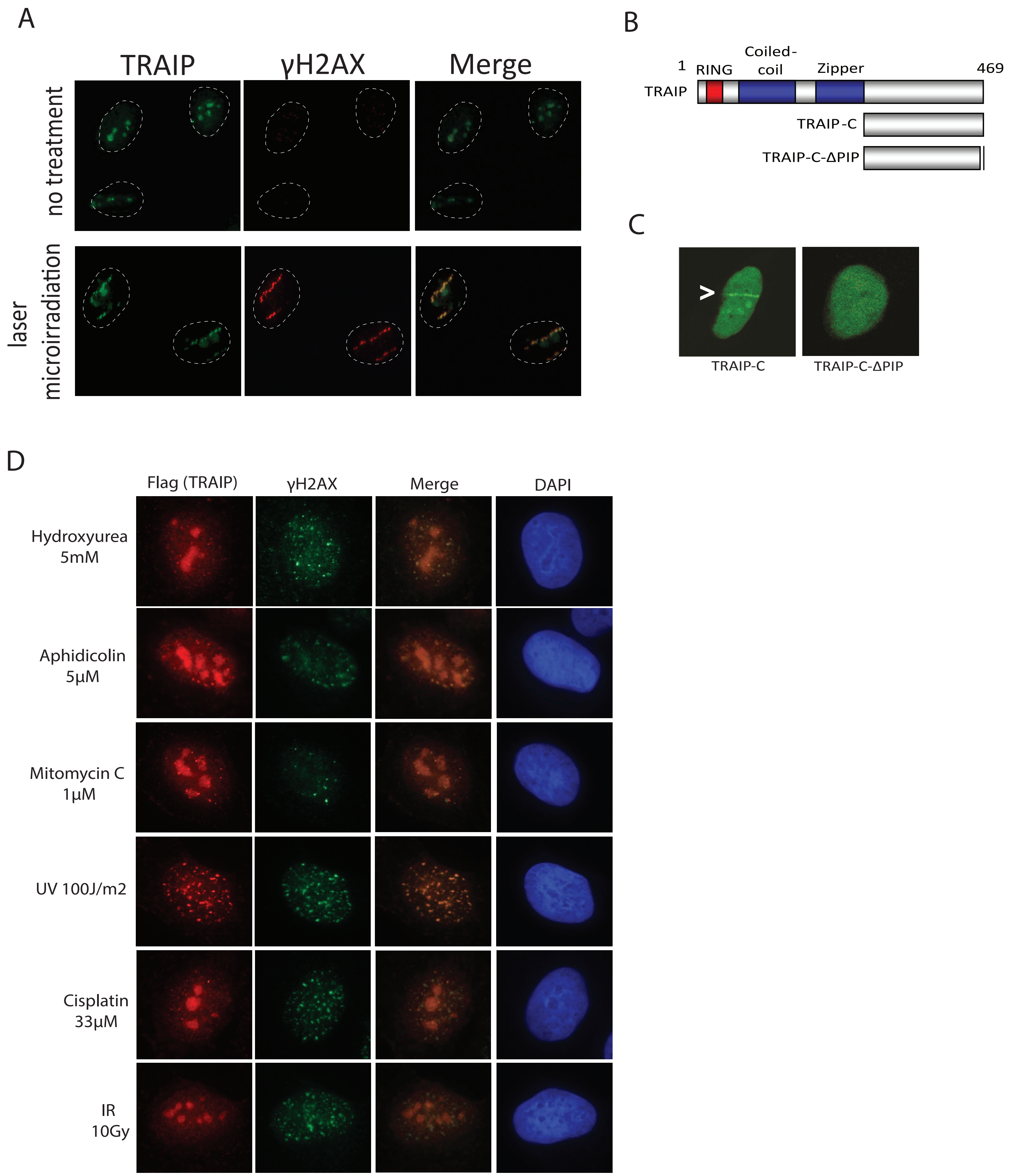

Supplementary Figure S3
A) U2OS cells subjected to laser micro-irradiation were fixed 1 hour after for indirect immunofluorescence staining experiments using indicated antibodies; B) Domain organization of TRAIP and its mutants; C) TRAIP-C, but not TRAIP-C-ΔPIP (PIP deletion) accumulated at laser-induced DNA damage tracks. Cells were subjected to laser micro-irradiation as in (A); D) U2OS cells engineered to stably express TRAIP-Flag were challenged with indicated DNA damaging agents. 4 hr post-incubation or IR treatment cells were processed for indirect immuno-fluorescence experiments using indicated antibodies. Nuclei were counter-stained with DAPI.
