# Supplementary material for: TRAIP regulates replication fork recovery and progression via PCNA
Source: Cell Discov. 2016 Jun 28;2:16016–. doi: 10.1038/celldisc.2016.16 (PMC4923944; doi:10.1038/celldisc.2016.16)
Supplement: Supplementary Figure S4 [file celldisc201616-s4.pdf]

# Supplementary Figure S4

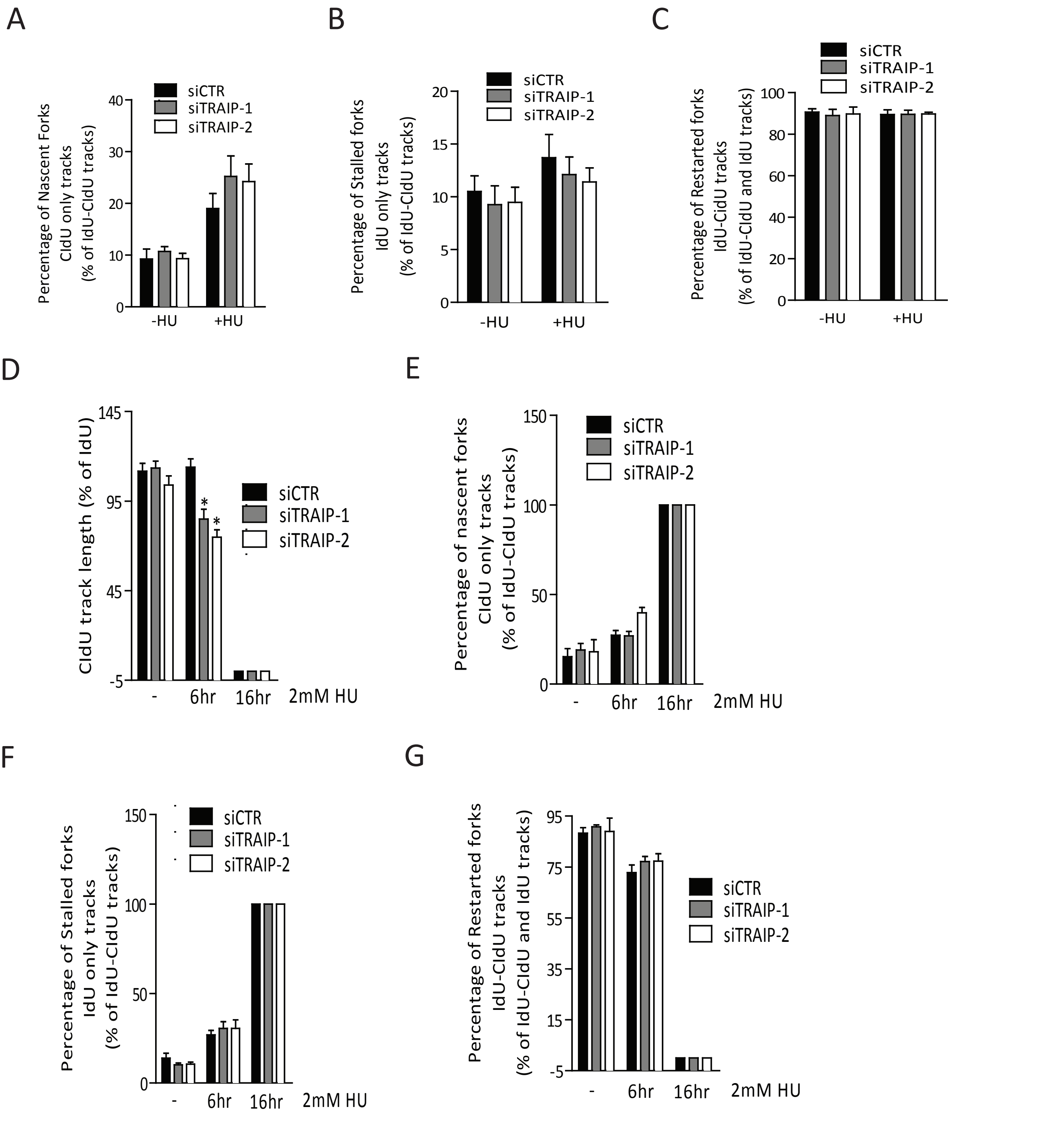


Supplementary Figure S4

A – G) DNA from cells pre-treated with TRAIP-specific siRNAs (siTRAIP-1 and siTRAIP-2) or control siRNAs (siCTR) were combed and immuno-labeled to determine fork progression with or without hydroxyurea (HU) treatment. As depicted in Figure 7A, U2OS cells were pulse-labeled with IdU, treated with 10 mM HU for 2 hours (A - C) or with 2mM HU for indicated time durations (D - F), and were subsequently released into fresh medium supplemented with CIdU. Number of nascent forks (A & E), stalled forks (B & F) and restarted forks (C & G) from control and TRAIP-depleted cells are shown. TRAIP inactivation compromised rate of restarted forks (D). CIdU track length is shown as percentage of IdU track length. Results represent mean±S.E.M. from three independent experiments and at least 250 structures were counted per experiment. Values marked with asterisks are significantly different from control. Statistical significance was assessed by Student's t-test (*p<0.05 vs. control).
