# Supplementary material for: TRAIP regulates replication fork recovery and progression via PCNA
Source: Cell Discov. 2016 Jun 28;2:16016–. doi: 10.1038/celldisc.2016.16 (PMC4923944; doi:10.1038/celldisc.2016.16)
Supplement: Supplementary Figure S5 [file celldisc201616-s5.pdf]

Supplementary Figure S5

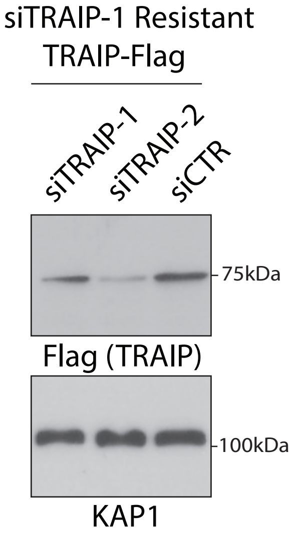

Supplementary Figure S5
293T cells were transfected with control siRNA (siCTR) or TRAIP-specific siRNAs (siTRAIP-1 or siTRAIP-2) together with expression construct encoding siTRAIP-1-resistant TRAIP-Flag. Western blotting analysis of TRAIP levels was performed using anti-Flag (M2) antibodies. KAP-1 was used as loading control.
