# Supplementary material for: TRAIP regulates replication fork recovery and progression via PCNA
Source: Cell Discov. 2016 Jun 28;2:16016–. doi: 10.1038/celldisc.2016.16 (PMC4923944; doi:10.1038/celldisc.2016.16)
Supplement: Supplementary Figure S6 [file celldisc201616-s6.pdf]

Supplementary Figure S6

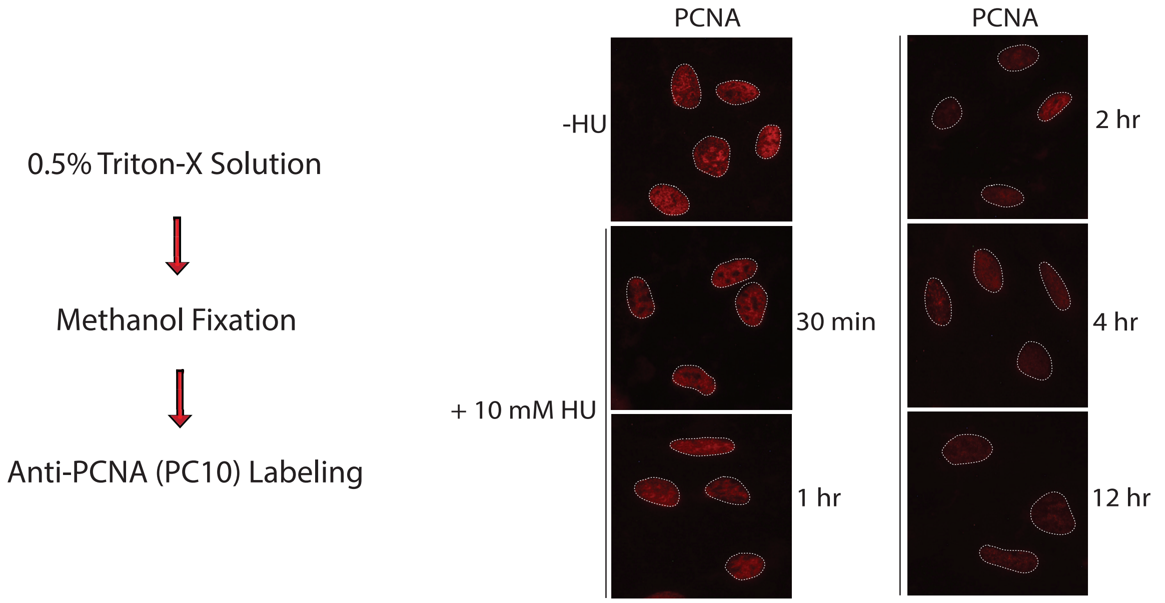

Supplementary Figure S6
U2OS cells were either left untreated or were incubated with 10 mM hydroxyurea (HU). At indicated time points cells were processed as described (left panel) to visualize subcellular localization of PCNA.
