# Supplementary material for: TRAIP regulates replication fork recovery and progression via PCNA
Source: Cell Discov. 2016 Jun 28;2:16016–. doi: 10.1038/celldisc.2016.16 (PMC4923944; doi:10.1038/celldisc.2016.16)
Supplement: Supplementary Figure S7 [file celldisc201616-s7.pdf]

Supplementary Figure S7

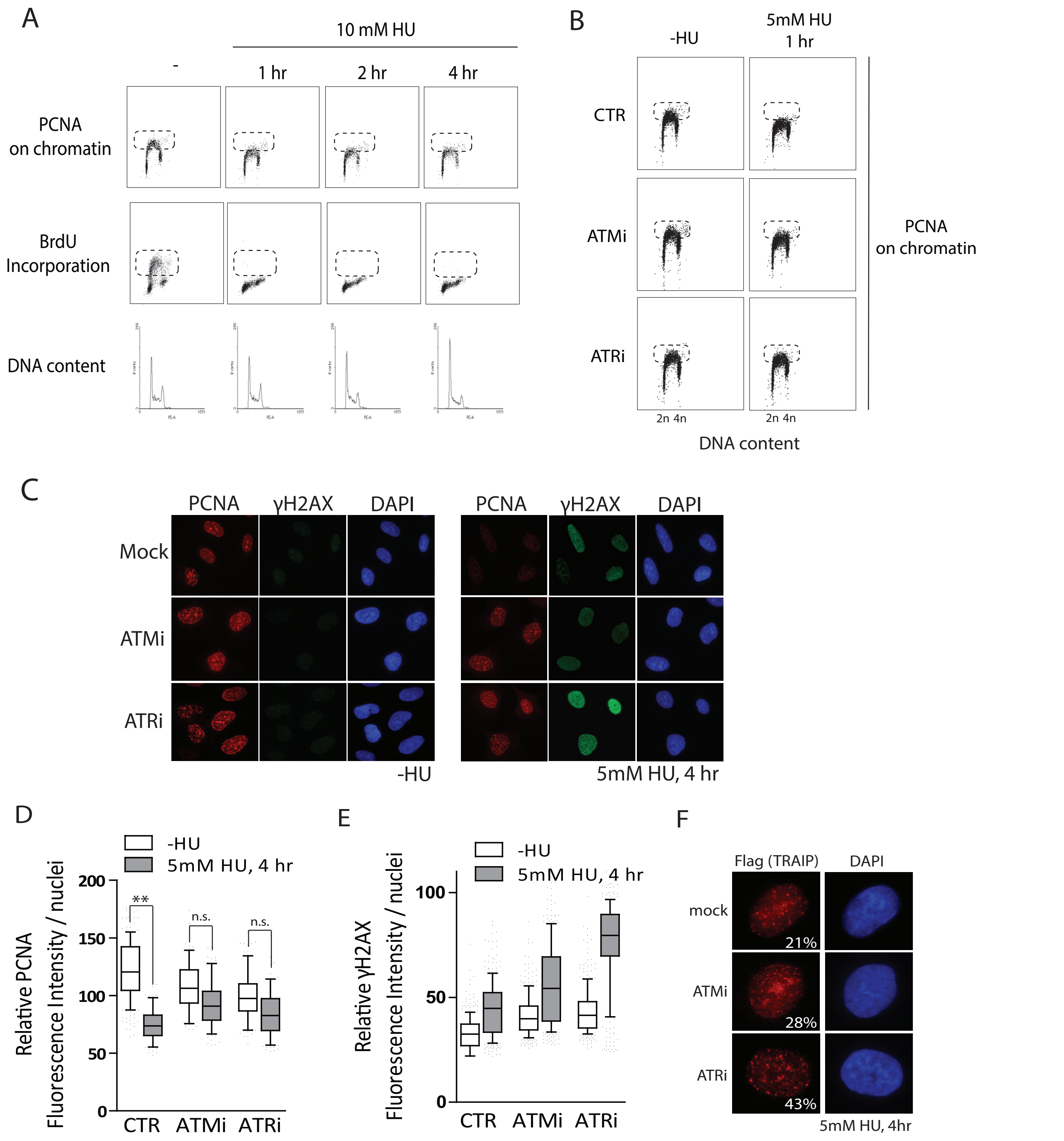

Supplementary Figure S7

A) U2OS cells were treated with hydroxyurea (HU) for indicated time durations and were subsequently pulse-labeled with 10 μM BrdU for 15 min. Cells were trypsinized, pre-extracted with 0.5% triton X-100 solution and methanol-fixed. Chromatin-bound PCNA was determined by immuno-staining with mouse monoclonal anti-PCNA antibodies (PC10). BrdU uptake, reflective of nascent DNA synthesis, was examined according to manufacturer's protocol. Total DNA content was determined by propidium iodide (PI) staining; B - E) U2OS cells pre-treated with ATMi (KU55993) and ATRi (VE821) were incubated with or without HU. Chromatin-bound fractions of PCNA were determined by flow cytometry analysis (B) and immunofluorescence experiments (C). γH2AX was used as a DNA damage marker. Fluorescence intensities of PCNA (D) and γ-H2AX (E) labeling were measured within the entire nuclei. Box & whiskers plots show the mean values of fluorescence intensities of > 100 cells from three independent experiments. Error bars represent S.D., **$p<0.05$ vs -HU controls; F) Cells expressing TRAIP-Flag were pre-treated with DMSO, ATMi (KU55993) or ATRi (VE821) prior to incubation with HU. Cells were subsequently processed for indirect immunofluorescence experiments using anti-Flag antibodies. Percentage of cells with TRAIP foci is shown.
