# Supplementary material for: TRAIP regulates replication fork recovery and progression via PCNA
Source: Cell Discov. 2016 Jun 28;2:16016–. doi: 10.1038/celldisc.2016.16 (PMC4923944; doi:10.1038/celldisc.2016.16)
Supplement: Supplementary Figure S8 [file celldisc201616-s8.pdf]

Supplementary Figure S8

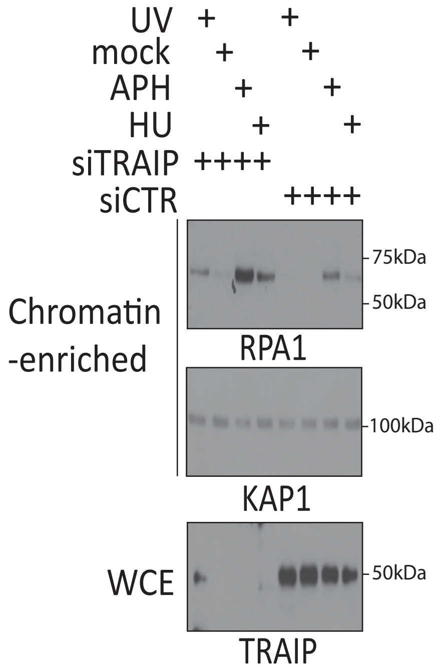

Supplementary Figure S8
U2OS cells pre-treated with control siRNA (siCTR) or TRAIP-targeting SMARTpool siRNAs (siTRAIP) were challenged with UV (100J/m2), aphidicolin (APH; 5µM), hydroxyurea (HU; 5mM) or left untreated (mock). 4 hours post-treatment whole cell extracts (WCE) and chromatin-enriched fractions were prepared and analysed by Western blotting experiments using indicated antibodies. KAP1 was used as loading control.
