# Supplementary material for: TRAIP regulates replication fork recovery and progression via PCNA
Source: Cell Discov. 2016 Jun 28;2:16016–. doi: 10.1038/celldisc.2016.16 (PMC4923944; doi:10.1038/celldisc.2016.16)
Supplement: Supplementary Figure S9 [file celldisc201616-s9.pdf]

Supplementary Figure S9

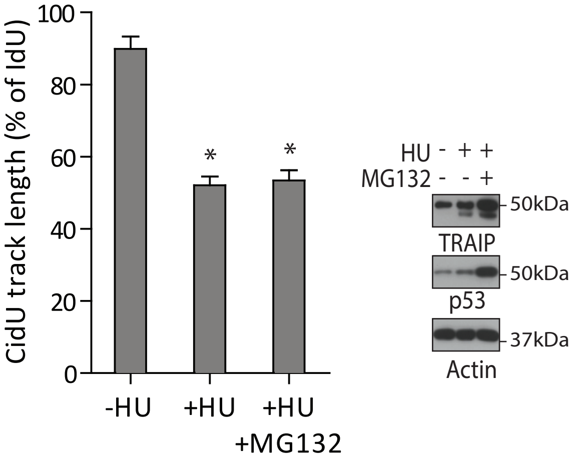

Supplementary Figure S9

U2OS cells were pulse-labeled with IdU, treated with hydroxyurea (HU; 10mM) with or without proteasome inhibitor MG132 (10 μM) for 2 hours, and released into fresh medium containing CIdU and MG132 (10μM). CIdU track length is shown as percentage of IdU track length. Results represent mean±S.E.M. from three independent experiments where at least 250 structures are counted for each experiment. Statistical significance was assessed by Student's t-test (*p<0.05). Corresponding U2OS cells were lysed and immunoblotting experiments were performed using indicated antibodies. p53 was used as an indicator to monitor proteasome inhibition following MG132 treatment.
