# Supplementary material for: TRAIP regulates replication fork recovery and progression via PCNA
Source: Cell Discov. 2016 Jun 28;2:16016–. doi: 10.1038/celldisc.2016.16 (PMC4923944; doi:10.1038/celldisc.2016.16)
Supplement: Supplementary Figure S10 [file celldisc201616-s10.pdf]

Supplementary Figure S10

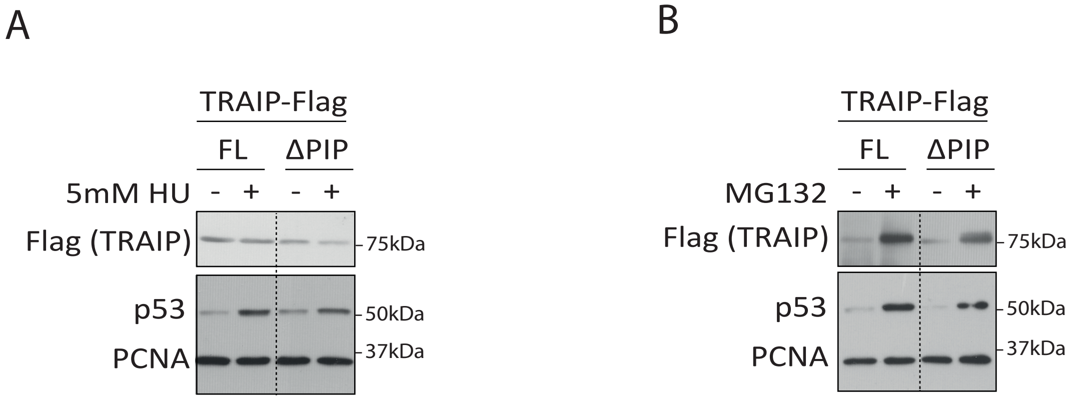

Supplementary Figure S10

A - B) TRAIP-Flag (full-length FL or its PIP deletion mutant; ΔPIP) expression level was determined by Western blotting experiment. Cells expressing TRAIP-Flag (or its PIP deletion mutant; ΔPIP) were harvested for Western blotting analysis to determine effect hydroxyurea (HU; panel A) and MG132 (B) on protein expression and stability. p53 and PCNA were used as positive and loading controls, respectively.
