# Supplementary material for: TRAIP regulates replication fork recovery and progression via PCNA
Source: Cell Discov. 2016 Jun 28;2:16016–. doi: 10.1038/celldisc.2016.16 (PMC4923944; doi:10.1038/celldisc.2016.16)
Supplement: Supplementary Figure S11 [file celldisc201616-s11.pdf]

Supplementary Figure S11

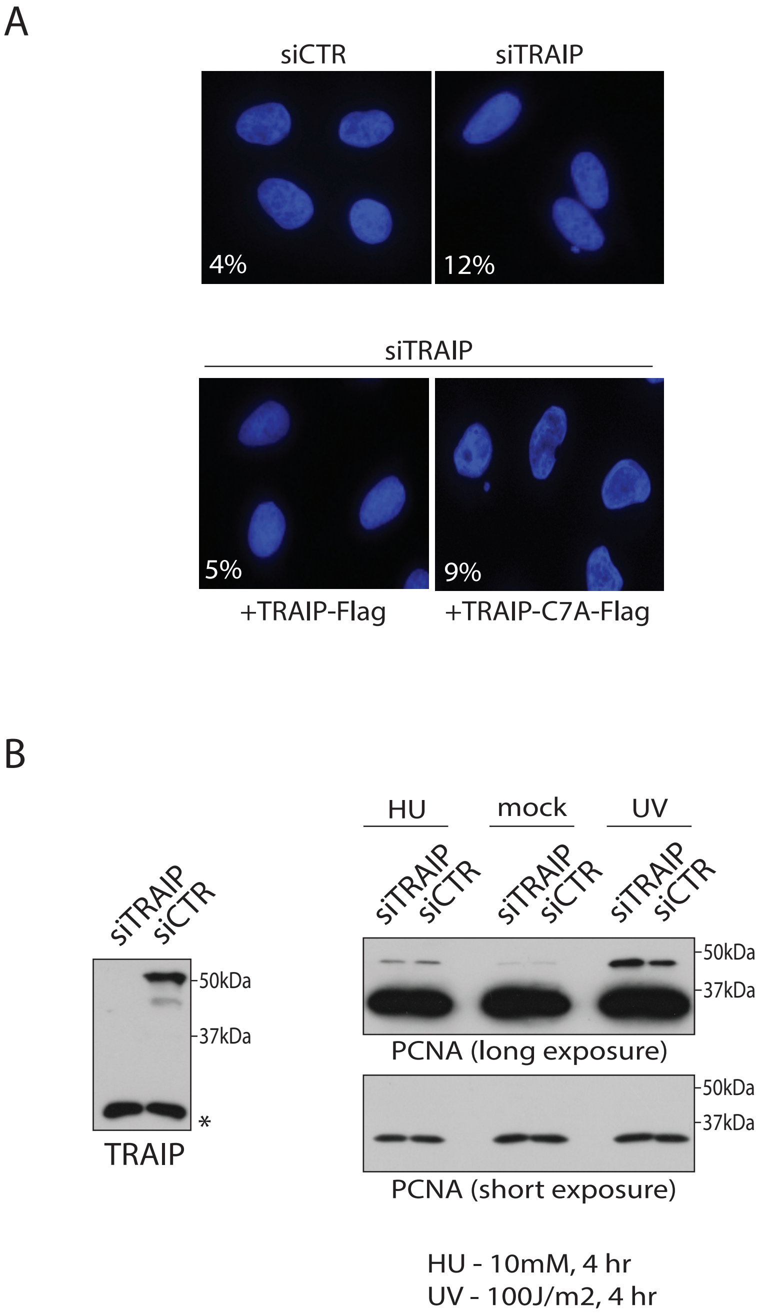

Supplementary Figure S11

A) Micronuclei formation was scored in U2OS cells pre-treated with control siRNAs (siCTR), TRAIP-targeting siRNAs (siTRAIP), or in TRAIP-depleted cells reconstituted with wildtype TRAIP or its RING mutant (C7A). Percentages of cells with micronuclei is shown and were derived from three independent experiments, n=100; B) U2OS cells pre-treated with indicated siRNAs were subjected to HU, UV or mock treatment. Cells were subsequently lysed for Western blotting experiments to determine PCNA expression and PCNA mono-ubiquitylation. Efficiency of TRAIP knockdown was assayed by anti-TRAIP labeling.
